# 3D Filaments Based on Polyhydroxy Butyrate—Micronized Bacterial Cellulose for Tissue Engineering Applications

**DOI:** 10.3390/jfb14090464

**Published:** 2023-09-09

**Authors:** Matheus F. Celestino, Lais R. Lima, Marina Fontes, Igor T. S. Batista, Daniella R. Mulinari, Alessandra Dametto, Raphael A. Rattes, André C. Amaral, Rosana M. N. Assunção, Clovis A. Ribeiro, Guillermo R. Castro, Hernane S. Barud

**Affiliations:** 1 Biopolymers and Biomaterials Group, Postgraduate Program in Biotechnology, University of Araraquara (UNIARA), Araraquara 14801-320, SP, Brazilitsbatista@gmail.com (I.T.S.B.); acamaral@uniara.edu.br (A.C.A.); 2 Institute of Chemistry, University of São Paulo (USP), São Carlos 13566-590, SP, Brazil; laisroncalho@gmail.com; 3 Biosmart Nanotechnology LTDA, Araraquara 14808-162, SP, Brazil; 4 Department of Mechanics and Energy, State University of Rio de Janeiro (UEJR), Rio de Janeiro 20550-900, RJ, Brazil; 5 Faculty of Integrated Sciences of Pontal (FACIP), Federal University of Uberlandia (UFU), Pontal Campus, Ituiutaba 38304-402, MG, Brazil; 6 Institute of Chemistry, São Paulo State University (UNESP), Araraquara 14800-900, SP, Brazil; 7 Nanomedicine Research Unit (Nanomed), Center for Natural and Human Sciences, Federal University of ABC (UFABC), Santo André 09210-580, SP, Brazil

**Keywords:** 3D printing, micronized bacterial cellulose, poly(hydroxybutyrate), tissue engineering, scaffolds

## Abstract

In this work, scaffolds based on poly(hydroxybutyrate) (PHB) and micronized bacterial cellulose (BC) were produced through 3D printing. Filaments for the printing were obtained by varying the percentage of micronized BC (0.25, 0.50, 1.00, and 2.00%) inserted in relation to the PHB matrix. Despite the varying concentrations of BC, the biocomposite filaments predominantly contained PHB functional groups, as Fourier transform infrared spectroscopy (FTIR) demonstrated. Thermogravimetric analyses (i.e., TG and DTG) of the filaments showed that the peak temperature (T_peak_) of PHB degradation decreased as the concentration of BC increased, with the lowest being 248 °C, referring to the biocomposite filament PHB/2.0% BC, which has the highest concentration of BC. Although there was a variation in the thermal behavior of the filaments, it was not significant enough to make printing impossible, considering that the PHB melting temperature was 170 °C. Biological assays indicated the non-cytotoxicity of scaffolds and the provision of cell anchorage sites. The results obtained in this research open up new paths for the application of this innovation in tissue engineering.

## 1. Introduction

Three-dimensional (3D) printing, also referred to as additive manufacturing (AM), has gained significant traction in the industry due to its ability to achieve mass customization and bring intricate designs to life, surpassing the limitations of traditional manufacturing methods [1]. Among the several 3D printing technologies, fused deposition modeling (FDM) stands out as the market’s most widely used method of obtaining scaffolds [2].

The FDM technique was introduced and commercialized by the Stratasys corporation in the United States during the early 1990s. Since then, FDM has been employed to produce several materials, ranging from polymers and metal powder to ceramics and composites. FDM has gained significant popularity in biomaterial research due to its affordability, compact size, ability to create intricate structures, and lack of organic solvents [3]. In FDM, materials are melted and deposited layer by layer onto a print bed, following a programmed pattern [4].

Polymers are the most used class of materials in additive manufacturing due to their high availability, diversity of applications, and low cost [5]. Nowadays, polymers from natural origins are preferred for 3D printing for tissue engineering because of their biocompatibility, in addition to being renewable, non-toxic, biodegradable, sustainable, and ecologically correct [6].

Currently, thermoplastics such as poly(lactic acid) (PLA) and acrylonitrile butadiene styrene (ABS) dominate the FDM materials market [7]. Due to its non-biodegradability and limited cell integration, ABS is not the preferred choice for fabricating tissue engineering scaffolds [8]. PLA is an example of a biodegradable and bioresorbable thermoplastic biopolymer that has been successfully applied in regenerative medicine and tissue engineering, being widely used for the construction of scaffolds via 3D FDM printing [9,10,11,12]. In addition, PLA gels can cause irritation via polymer hydrolysis in the surrounding tissue in which they are applied because the *pKa* of lactic acid is 3.86.

A class of biopolymers that has gained prominence in recent years is polyhydroxyalkanoates (PHAs). PHAs are synthesized directly from bacterial metabolism occurring at low concentrations of nitrogen, phosphorus, oxygen, or magnesium and an excess of carbon, representing an advantage compared to PLA. Other advantages are their complete biodegradability and the multiplicity of their structures [5,9]. Poly(hydroxybutyrate) (PHB) is an extensively investigated member of the polyhydroxyalkanoate (PHA) family. It is a biodegradable biopolymer with piezoelectric properties, rendering it suitable for various biomedical applications. PHB demonstrates high biocompatibility with different cell types, including osteoblasts, epithelial cells, and chondrocytes. Its biocompatibility is attributed to the presence of low molecular weight PHB in the body and the natural occurrence of its degradation product, 3-hydroxybutyric acid, which serves as a natural metabolite in organs like the brain, heart, and lungs. These inherent characteristics, coupled with its ability to promote bone growth, favorable mechanical properties, and cost-effectiveness, position PHB as a highly promising material for medical applications [13,14].

Although it has desirable characteristics for a wide spectrum of applications, PHB has some shortcomings that limit its use for producing the scaffolds applied in tissue engineering, especially if the FDM technique is used; these shortcomings include hydrophobicity, a low degradation rate, fragility, and contamination via pyrogenic compounds and thermal instability [13,14]. To address the limitations of PHB, the incorporation of PHB into composites with other biopolymers has been extensively explored [15,16,17]. In particular, cellulose has emerged as a commonly used biopolymer for this purpose. In a study by da Silva Moura et al. (2019) [18], treated coconut fibers containing approximately 40–60% cellulose were employed as reinforcing agents in PHB composites. Including these fibers resulted in enhanced mechanical properties, improved thermal stability, and an increased modulus of elasticity without additional additives. Similarly, Barud et al. (2011) [19] utilized bacterial cellulose (BC) to fabricate composites, which exhibited superior mechanical properties compared to the individual polymers, mainly in terms of tensile strength, elongation to rupture, and Young’s modulus.

BC is composed of β-D-glucopyranose units joined together by β-1,4-glycosidic bonds arranged in a ribbon-like network of fibrils less than 100 nm in length and 2–4 nm in diameter. Unlike vegetal cellulose, the BC network is free of lignin, hemicellulose, and other constituents of lignocellulosic materials [20]. In addition to the composition, BC differs from celluloses from other sources due to its high degree of purity and polymerization (up to 8000), crystallinity (70–80%), high water content (up to 99%), physical and mechanical resistance, flexibility, and high biocompatibility [21]. BC is a non-cytotoxic, non-genotoxic, biodegradable, and biocompatible biomaterial [22,23]. This interest is reflected by the variety of works in the literature involving bacterial cellulose, many of them focused on medical applications and, more specifically, in the tissue engineering sector [24,25,26,27].

The high applicability of BC is not only due to its unique characteristics but also to the forms and structures employed. BC can be used in the form of a membrane, either wet or dry, in the form of cellulose nanomaterials (CNM), such as cellulose nanocrystals (CNC), cellulose nanofibers (CNF), obtained through the rupture of amorphous domains or from the simple separation of fiber bundles, and as micronized cellulose particles (CMP), obtained through treatments capable of weakening supramolecular interactions through a process called cellulose activation [28]. However, there are challenges to be faced when producing high-quality BC (and its derivatives), such as the reduction of operating costs and the viability of large-scale production. For this reason, there has been a growing interest in the search for solutions, such as developing new bioreactor projects and process automation. Furthermore, alternative raw materials (especially waste) have been explored as a promising option [23,29].

MELO et al. (2020) [30] demonstrated the circular economy for BC by recycling its waste from commercial wound dressings to obtain CNC and develop sustainable and biodegradable packaging. An additional example of the circular economy in action in the production chain of BC and its derivatives is the replacement of hazardous chemical processes and/or reagents commonly used in BC pre-treatment and hydrolysis to obtain CNM and CMP through processes and/or sustainable reagents. Swelling agents such as dimethylsulfoxide (DMSO) and dimethylformamide (DMF) are often used to activate cellulose and obtain micronized particles. However, these reagents pose risks to human health and are not ecologically friendly, in addition to requiring high financial and energy costs for production, purification, collection, recycling, and disposal. Therefore, there is a growing search for sustainable alternatives, such as mechanochemical processes, to obtain these structures [31].

In this context, aiming at the concept of upcycling and circular economy, the present work developed a biocomposite product with high added value, such as scaffolds. Micronized BC was obtained from industrial dressing residues through the mechanochemical process, and was used as a reinforcing agent in the development of biocomposite filaments based on a PHB matrix. The manufactured scaffolds were physicochemically and morphologically characterized, and in vitro cytotoxicity assays were performed to confirm the viability for tissue engineering applications.

## 2. Materials and Methods

### 2.1. Materials

The PHB, Biocycle 1000 (Mn 147.596 g mol^−1^, Mw 376 g mol^−1^, and PDI 2.5), was supplied by PHB Industrial S/A, São Paulo. Bacterial cellulose residues were provided by the company BioSmart Nanotechnology LDTA. The micronizes were obtained using a Polymix^®^ PX-IG 2000 Impact Grinder ball milling from the company Kinematica. The mechanochemical process was applied for 20 min at frequencies of 10 Hz, 20 Hz, and 30 Hz.

To obtain the biocomposites, micronized BC at 20 Hz was used. Both PHB and BC biopolymers were submitted to a thermokinetic mixer (MH-50H, 48 A), with speed maintained at 5250 rpm, for 1 min. The amount of micronized BC inserted into the PHB varied between 0.25%, 0.50%, 1.00%, and 2.00% (wt%). The mixtures obtained were ground in a granulating mill (Plastimax, 3.7 kW) and dried at 50 °C for 2 h. The biocomposites were extruded in a mini extruder (Weellzoom, model B Desktop, Guangdong Province, Guangzhou, China) to obtain the filaments. The temperature in processing the filaments was 165 °C, and the extrusion speed was 85 mm min^−1^.

### 2.2. Methods

X-ray diffraction (XRD) measurements were performed on an XRD-6000 diffractometer (Shimadzu). The patterns of micronized BC were recorded using Cu-Kα radiation (λ = 1.5406 Å) at 40 kV and 40 mA in the 2θ region from 10 to 60°. Segal and peak deconvolution methods were applied to diffractograms to analyze the influence of micronization on crystallinity indices (CI). The pseudovoight 1 function was used to determine the crystalline portion from peak deconvolution. The particle size of BC was determined using the Anton-Paar PSA 1190 LD particle size analyzer. The results were reported for D10, D50, and D90, which are the volume diameters of the particles at 10%, 50%, and 90% cumulative volume, respectively. The surface area was measured using the Anton-Paar NOVA touch BET Surface Area and Pore Size Analyzers, with nitrogen as the adsorbate. The degree of polymerization (DP) of BC was determined using the transparent Cannon-Fenske-type viscometer n. 150, according to TAPPI standard T 230om-94: Viscosity of pulp (capillary viscometer method), 2013. Time measurements were performed in triplicate and the average obtained was used to calculate the degree of polymerization following the calculations presented by [32].

BC surface and filament cross-section morphologies were visualized in a JEOL 7500F electron microscope at 2.00 kV after being fixed onto stubs using a carbon film and coated with a thin carbon layer. Thermal analysis of both BC and PHB/BC biocomposite filaments was performed using TA Instruments SDT Q600 equipment. The samples were heated in an alumina crucible from 30 to 600 °C within an atmosphere of N_2_ flowing at 100 mL min^−1^. Fourier Transform Infrared Spectroscopy was performed using a Bruker-Vertex 70 spectrophotometer in attenuated total reflection (ATR) mode. The BC and PHB/BC biocomposites spectra were obtained by accumulating 64 scans with a 2 cm^−1^ resolution in the range of 4000–650 cm^−1^.

For the 3D printing of the scaffolds, the digital design was taken from the “Thingiverse” file bank. Design sizing and slicing were performed using Ultimaker Cura 4.0 software. The scaffolds were sized 10 × 10 × 5 mm on the x, y, and z axes, respectively, and the part fill was set to 50%. Printing was performed using a CREALITY Ender-3 3D printer, with a maximum speed of 180 mm s^−1^, 0.4 mm nozzle, structure in anodized aluminum, and printing area of 220 × 220 × 250 mm. The temperature used in the process was 185 °C.

For PHB/BC biocomposite filament cell viability assays, L929 cells were cultured at 1 × 10^4^ cells/well in a 96-well culture plate. The cell viability was performed via MTT (3-(4,5-dimethylthiazol-2-yl)-2,5-diphenyltetrazolium bromide) assay, according to International Organization of Standardization (ISO) protocols for the biological evaluation of medical devices [33,34]. Thus, filaments from polymer blending between PHB and BC named PHB/BC 0.25%, PHB/BC 0.50%, PHB/BC 1.00%, and PHB/BC 2.00% were cut into 6 cm size and incubated with 3 mL of DMEM extraction medium at 37 °C for 24 h. Subsequently, the cell culture medium was removed, and 100 µL of each extraction media was added in contact with monolayer culture for 24 h in a CO_2_ incubator. Afterward, the extract media were removed, and the wells were washed three times with PBS 1X. Next, 100 µL of the MTT solution (1 mg mL^−1^) was added to each test well. Further, the microplate was incubated at 37 °C for 3 h. After the MTT was removed and 50 µL of isopropanol added to each well, the optical density was read at a wavelength of 570 nm using a spectrophotometer reader (SoftMax^®^ Pro 5). The assay was performed in triplicate. Cell viability was defined as the absorbance ratio from the sample to the absorbance measured for negative control (survive control) and represented as a mean value ± standard deviation [35].

To confirm the PHB/BC-based biocomposite as a scaffold, cell adhesion assays were performed. PHB/BC scaffolds were seeded with 5 × 10^4^ cells/well in a 24-well culture plate containing 2 mL of DMEM supplemented with 10% FBS and 100 U/mL penicillin-streptomycin and maintained at 37 °C in a CO_2_ incubator for 3 days to study cell attachment. The cultured cells on the scaffolds were then fixed with glutaraldehyde 0.25% for 0.5 h and then washed three times with PBS 1X. Afterward, the scaffolds were dehydrated with ethanol series and air-dried. Scanning Electron Microscopy (SEM) micrographs were captured under a JEOL JSM-6510 operating at 5 kV. All specimens were sputter coated with gold (Balzer, SCD 050) [36].

## 3. Results

### 3.1. Bacterial Cellulose Micronized

**Figure 1:**
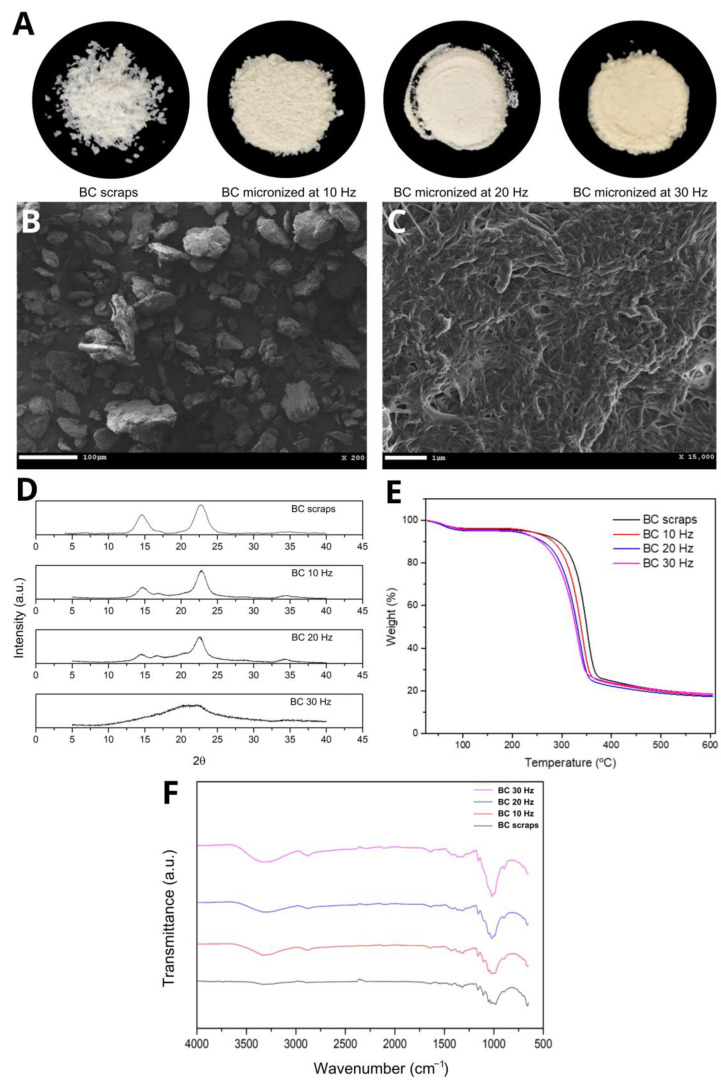
(**A**) Images of BC scraps and micronized BC at 10, 20, and 30 Hz, respectively; (**B**,**C**) representative SEM images of micronized BC at 20 Hz; (**D**) DRX curves, (**E**) TGA curves and (**F**) FTIR spectra of BC and micronized BC scraps at 10, 20, and 30 Hz, respectively.

The micronized BC resulting from the mechanochemical process at three different grinding frequencies (10, 20, and 30 Hz) had a powdery appearance and a slightly yellowish color when micronized at 30 Hz. This coloration is possibly due to the rise in the temperature of the process, consequent to the increase in frequency and, therefore, the increase in energy caused by the greater collision of the sample in the ball mill (Figure 1A). Scanning Electron Microscopy (SEM) showed that despite the milling at frequencies 10 and 20 Hz, the BC fibers were maintained compared to the BC before the mechanochemical process. Figure 1B shows a representative image of micronized BC at 20 Hz and Figure 1C confirms that the fibers morphology was maintained. It was also observed that increasing the grinding frequency to 30 Hz disfigured the cellulose fibers, forming agglomerates and exposing more of the amorphous portion of the cellulose.

The mechanochemical method known as ball milling is widely disseminated in the literature and used to obtain many micronized products [37,38,39,40]. This technique is used to fragment the BC into microparticles through mechanical collisions that break the hydrogen bonds, which are responsible for most of the intra- and intermolecular bonds and, consequently, for the three-dimensional crystalline structure of this biopolymer [31,41,42].

For the development of biocomposite filaments, the micronized particles must present an adequate balance between the characteristics of crystalline and amorphous cellulose structures since BC is used as a reinforcing agent. Still, it also needs to be degraded by the organism as cells differentiate and tissue regenerates.

Crystallinity indices (*CI*) were determined by Segal and peak deconvolution methods. The equation used for the Segal method was:

$$CI=\frac{\left(I_{200}-I_{am}\right)}{\begin{array}{c} I_{200} \end{array}}$$

where *CI* is the relative crystallinity index, *I*_200_ is the maximum intensity (in arbitrary units) of the peak referring to the plane (200), and *I_am_* is the diffraction intensity of the amorphous portion at 2θ = 18°.

The pseudovoight 1 function was used to determine the crystalline portion via peak deconvolution. Through this function, it was possible to determine the peaks referring to the crystalline portion and the amorphous portion. Consequently, the amounts of crystalline and amorphous material in the micronized BC samples were determined. This method considers the contributions of amorphous and crystalline cellulose for the entire spectrum; therefore, this technique has greater precision for determining the *CI* than the Segal method [43]. The *CI* of micronized BC samples and BC residue, calculated using the diffractograms in Figure 1D, are shown in Table 1.

It is observed that the Segal method provides higher *CI* values than the peak deconvolution method, except for the micronized *CI* at 30 Hz. These values portray an overestimation of the *CI* as previously reported [43], and reinforce the results obtained by [42,44]. It is also noted that the BC micronized at 10 Hz maintained most of its crystalline structure. In comparison, the BC micronized at 30 Hz showed the dominance of the amorphous portion, probably due to the disruption of the intra- and intermolecular bonds. The BC micronized at 20 Hz presented a greater balance between the portions, which is favorable for obtaining the biocomposite.

The TGA curves (Figure 1E) show two events with considerable mass losses observed in all micronized BC samples. The first event between 50 and 150 °C (≈5% initial mass loss) is attributed to water loss. The second event between 250 and 400 °C (with a loss of ≈75% of the initial mass) corresponds to the thermal degradation of cellulose, i.e., the processes of depolymerization, dehydration, and decomposition of the glycosidic units followed by the formation of carbonaceous residues [45]. Cellulose micronization breaks the hydrogen bonds that maintain the three-dimensional crystalline structure, causing the cellulose chains to depolymerize and become amorphous, which corroborates the XRD results. Increasing the frequency of the mechanochemical process increased the entropy of the system, consequently leading to a decrease in decomposition temperatures [31]. That is, the depolymerization and amorphization phenomena are incremented with the increase in the milling frequency; therefore, the T_peak_, T_onset_, and T_offset_ temperatures are lower, and mass loss events are anticipated.

In the FTIR spectrum for BC scraps and their micronized counterparts (Figure 1F), the presence of significant absorption bands at the same wavelength was observed, indicating similarity between the functional groups, i.e., there was no evidence of the formation of new bonds or changes in chemical structure. It was also observed that as the grinding frequency of the scraps increased, the transmittance of the bands decreased, which was very evident in the bands at 3300 cm^−1^ referring to OH stretching, and at 1140–1015 cm^−1^ referring to deformation CO [46]. This can be explained by the decrease in the crystallinity of BC, while micronization breaks hydrogen bonds [47]. This effect was also observed by [48,49,50] in their respective works. The other absorption characteristic bands for BC are: 2880 cm^−1^—CH stretching of alkanes and asymmetric stretching CH_2_; 1645 cm^−1^—OH strain; 1420 cm^−1^—CH_2_ deformation; 1370 cm^−1^—OH deformation [46].

The results of the granulometric analysis (Table 2) demonstrated an inversely proportional relationship between the grinding frequency and the average diameter of the micronized BC. As the frequency in the mechanochemical process increased, the average particle diameter decreased. This relationship can be observed in the three measurement ranges, named D10, D50, and D90, which represent 10, 50, and 90% of the total volume of particles, respectively. Ball milling reduced the mean particle diameter, but standard deviation variations in percentiles were observed. At the frequency of 10 Hz, particles were reduced, but not all of them were homogeneous; therefore, the observed standard deviation was higher than the deviations of the other frequencies used. At the frequency of 30 Hz, an increase in standard deviation was also observed in the deviation at 20 Hz. This result is due to the agglomerative phenomena that occur in more energetic processes in which the portion amorphous part of the material is gradually more exposed [47,51,52,53]. In this case, the phenomena of breakage and aggregation co-occur.

The average diameter of the micronized BC and its respective standard deviations are fundamental parameters for its application in filament formation. The sizes directly interfere with the ability to obtain a homogeneous biocomposite and the ability of this material to be extruded uniformly without breaking, preventing clogging of the extruder heads and the 3D printer.

The micronized BC at 10 Hz showed a high diameter and standard deviation compared to the others, which could become an obstacle to obtaining and printing the filament. The micronized filaments at 20 and 30 Hz had similar diameters, but the standard deviation of the micronized filaments at 20 Hz was smaller; therefore, the micronized ones at 20 Hz could provide better homogeneity to the filaments.

In the analysis of the determination of the surface area, it was expected that the increase in the grinding frequency correlated with an increase in the observed surface area, following the results of the granulometric analysis and the results obtained by [44]. This relationship was observed with the frequencies 10 and 20 Hz. However, at the frequency of 30 Hz, the surface area decreased compared to the milling performed at lower frequencies (Table 3), possibly due to the aggregation of micronized products due to the high level of energy transferred in the process.

The degree of polymerization (DP) refers to the number of repeated structural units observed in the constitution of a macromolecule; that is, the number of monomers that constitute the polymer. As the frequency used in the micronization process increased, bond breaking also increased and, consequently, the DP decreased (Table 4). These results corroborate the literature, in which micronization processes tend to reduce the DP of polymers due to the breaking of the bonds that unite the monomers [54,55], in the case of BC, corresponding to the β-1,4 glycosidic bonds.

### 3.2. PHB/BC Biocomposite Filaments

The filaments obtained after extrusion were homogeneous, and no cracks, flaws, burns, or BC agglomerates were observed. All filaments showed relative fracture resistance, but the filaments without BC, with 0.25 and 0.50% BC were more malleable and flexible than the others.

**Figure 2:**
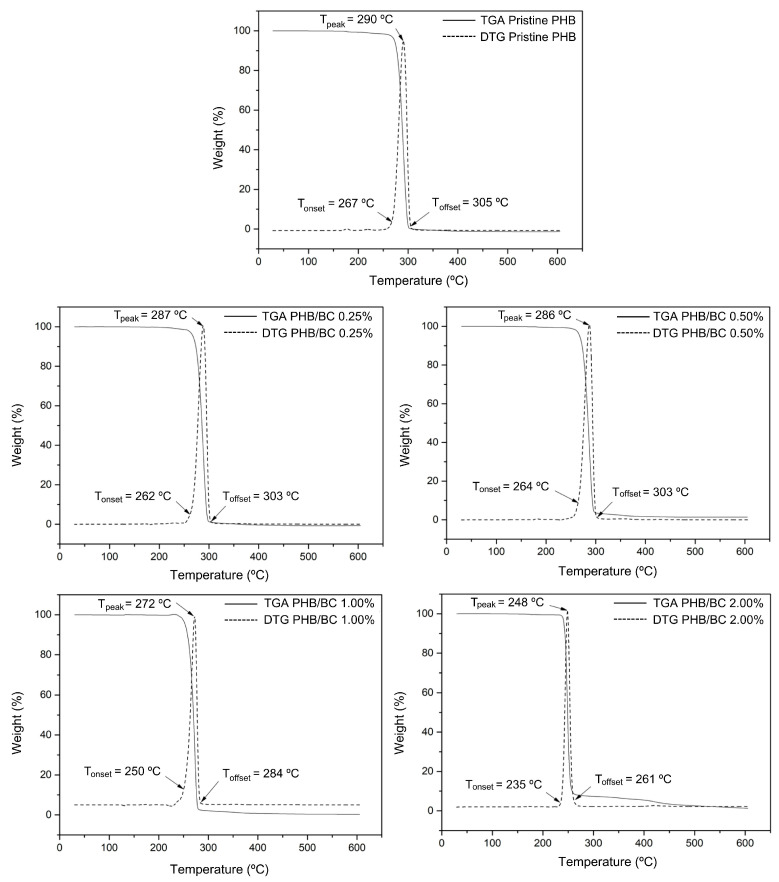
TGA and DTG curves of pristine PHB and PHB/BC-based biocomposite filaments containing 0.25, 0.50, 1.00, and 2.00% BC, respectively.

Figure 2 shows the TGA and DTG curves of the biocomposite filaments. Two mass loss events were observed in all samples. In the first event, between 50 °C and 120 °C, a small mass loss corresponds to the vaporization of residual moisture. In the second event, between 170 °C and 350 °C, the mass loss is significant, as complete degradation of PHB occurs, mainly due to the β-cleavage of the PHB chains in C=O and C–O bonds, which facilitates the formation of crotonic acid, dimeric, trimeric, and tetrameric volatiles [19,56,57]. PRADHAN et al. (2017) [56] also observed that the PHB synthesized from the fermentation of hydrolysates rich in hexose had greater crystallinity and resistance to thermal degradation. In addition, it is possible to observe in the TGA and DTG curves that the filaments with higher concentrations of BC showed a reduction in thermal resistance. The biocomposite filament PHB/2.00% BC, with the highest concentration of micronized BC, showed the lowest thermal resistance. This reduction in thermal resistance is related to the decrease in crystallinity of the filament as the micronized material was added. The amorphous portion of BC is exposed with the micronization of cellulose and consequent breakage of hydrogen bonds, and the three-dimensional crystalline structure responsible for thermal resistance is lost.

Considering that the filaments obtained are intended for application in scaffolds, the observed decrease in thermal stability does not represent a problem for application in 3D printing because the average melting temperature (T_m_) of PHB (temperature used in printing) is 170 °C and the lowest T_onset_ observed was 235 °C.

**Figure 3:**
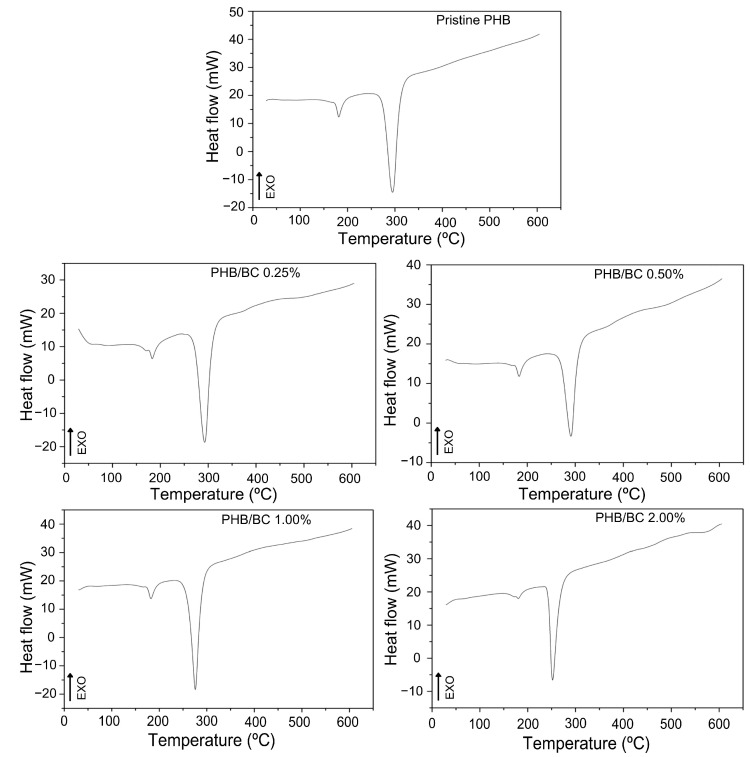
DSC curves of pristine PHB and PHB/BC-based biocomposite filaments containing 0.25, 0.50, 1.00, and 2.00% BC, respectively.

The DSC curves of the filaments (Figure 3) show two peaks of endothermic events at approximately 180 °C and 290 °C, which correspond to the melting and decomposition of PHB, respectively [19,56,58]. The DSC peak between 250 and 300 °C refers to the thermal decomposition of PHB. What is being observed is that the thermal stability of PHB decreases with the addition of micronized BC. Considering only PHB, the temperature of thermal decomposition depends on the number average molecular weight, Mn, and the weight average molecular weight, MW, and polydispersibility, PDI. PHB has a high PDI (2.5), which can contribute to decreased thermal stability. The literature presents the T_m_ of PHB close to 170 °C, but the T_m_ of PHB and its composites depend on many factors, such as morphology and particle size, crystallization kinetics, and the composition process [58]. PRADHAN et al. (2017) [56] obtained PHB through *Bacillus megaterium* and *Cupriavidus necator* with T_m_ observed at 175 °C and 176 °C, respectively; these values were also higher than the standard literature presents.

In Figure 3, TGA/DTG curves of PHB/BC, the T_onset_ and T_offset_ values were indicated. The increase in BC up to 2.0% in the PHB/BC composite decreased the maximum de-composition temperature (MDT) from 290 °C to 248 °C (∆T = 42 °C). However, BC concentrations up to 0.5% do not show drastic changes in the MDT, but high BC concentrations than 0.5% decrease the MDT in −25.1 °C/BC (%) (Figure S1, Supplementary Material) with consequent partial loss of PHB/BC composite stability.

Comparative analysis of TGA/DTGA with DSC shows some differences in the maximum endothermic temperatures compared with the MDT. However, it is possible to observe at the beginning of the reaction T_onset_, similar values in the DSC and TGA/DTG curves. Additionally, the trends of temperature decrease with the increase in BC content are similar in Figure 2 and Figure 3. Also, it is relevant to consider that during the processes of sample heating and cooling, TGA instruments are measuring the mass changes with the temperature; meanwhile, DSC measures the shifts in energy absorption or release related to the temperature variations.

In fact, TGA/DTG and DSC were obtained using the same equipment simultaneously and separated in Figure 2 and Figure 3 to facilitate the visualization of the events.

**Figure 4:**
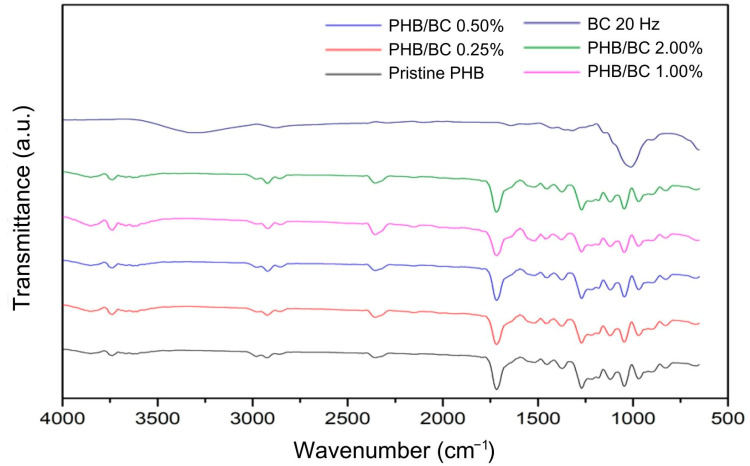
Fourier Transform Infrared Spectroscopy (FTIR) of micronized bacterial cellulose (BC) at 20 Hz, pristine poly(hydroxybutyrate) (PHB) filaments, and PHB/BC-based biocomposite filaments.

Figure 4 shows the main bands observed in the PHB/BC biocomposite filaments. Referring to PHB: 1000–1300 cm^−1^—C-O elongation of the ester group; 1455 cm^−1^—asymmetric bending of -CH_2_ or -CH_3_; 1718 cm^−1^—ester C=O elongation; 1271 cm^−1^—CH group; 2853 cm^−1^, 2926 cm^−1^ and 2981 cm^−1^—C-H elongation [59,60]. Regarding the micronized BC, the bands are different compared to the PHB. However, these vibrations were not observed in the spectra of the biocomposite filaments that remained with most of the individual characteristics of the PHB, probably because of the low sensitivity of the technique. This demonstrates that even when using micronized BC as a reinforcing agent, maintaining the PHB chemical groups in most of the biocomposite material was possible.

**Figure 5:**
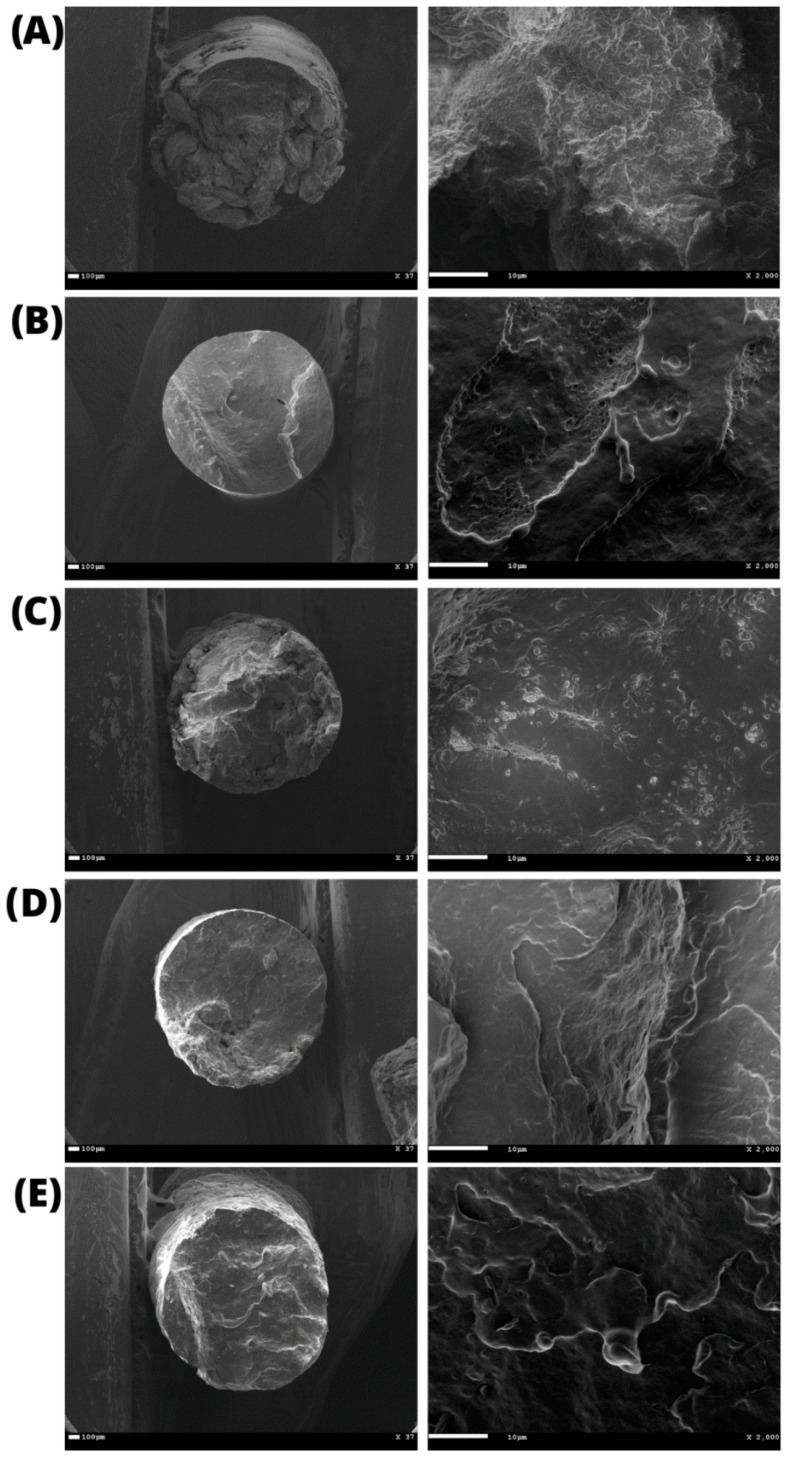
Scanning Electron Microscopy (SEM) of the printed filaments: (**A**) pristine PHB and PHB/BC-based biocomposites with 0.25, 0.50, 1.00, 2.00% BC ((**B**–**E**), respectively).

The average diameter of the filaments was 1.60 ± 0.04 mm, as determined by the images obtained by SEM (Figure 5). It was also possible to observe that the cross-section contained an entirely uniform, compact, and bubble-free surface, indicating high homogeneity, an essential characteristic for filaments used in 3D printing.

**Figure 6:**
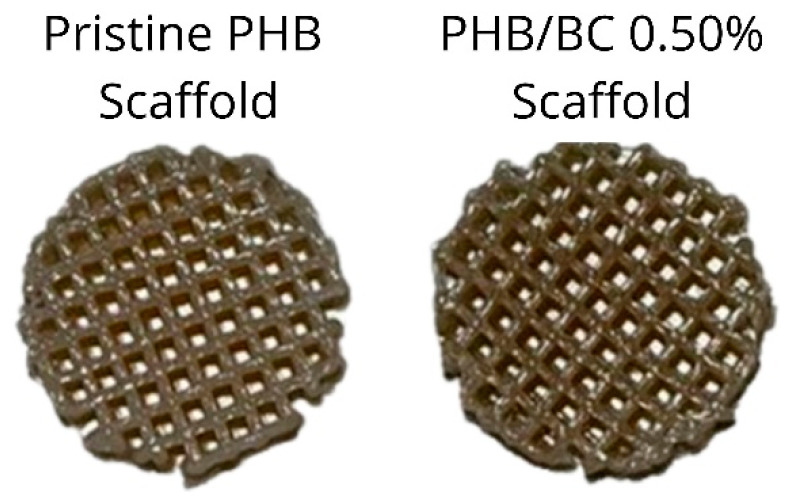
Scaffolds printed using pristine Poly(3-hydroxybutyrate) (PHB, **left**) and a representative image of PHB/0.50% BC-based biocomposite filaments (**right**).

The pristine PHB filaments and the PHB/BC-based biocomposites containing 0.25, 0.50, and 1.00% BC were printed homogeneously without clogging the printer head or breaking the filament during printing. Figure 6 shows a representative image comparing pristine PHB and PHB/0.50% BC-based scaffolds. Filaments with 2.00% of micronized BC caused clogging of the 0.4 mm printer nozzle diameter. This clogging probably occurred due to the cellulose particles aggregation on the surface of the filament and the consequent change in the viscosity of the material, as previously reported [61].

### 3.3. Biological In Vitro Assays

**Figure 7:**
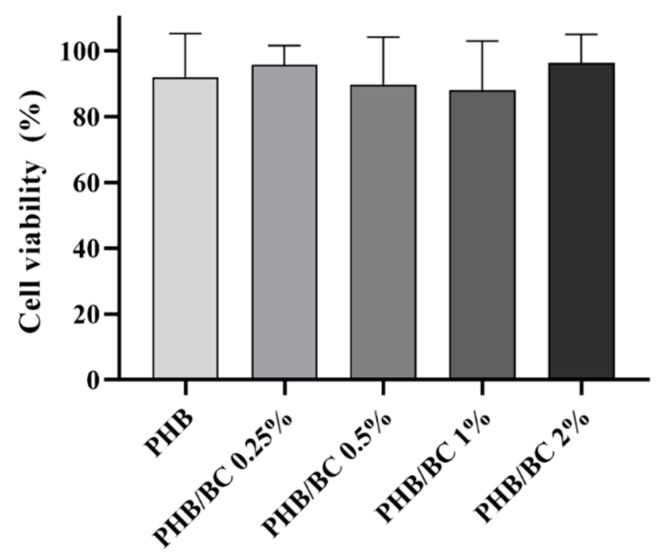
L929 viability assayed with the MTT assay revealed the using liquid extracts derived from PHB/BC-based biocomposites after 24 h of incubation.

The metabolic viability of L929 cells exposed to PHB/BC-based biocomposites was determined via the MTT assay after 24 h of exposure. The results showed that all samples were safe, resulting in cell viability higher than 70%, similar to control (Figure 7). This result confirmed previous ones that demonstrated no toxicity of biocomposites based on PHB/BC, and PHB blended with different biopolymers, including PLA and Hydroxyapatite (HA) [62,63]. Furthermore, the PHB/BC-based biocomposites proved to support 3T3-L1 preadipocyte proliferation and induced a positive effect on osteoblast differentiation in vivo.

**Figure 8:**
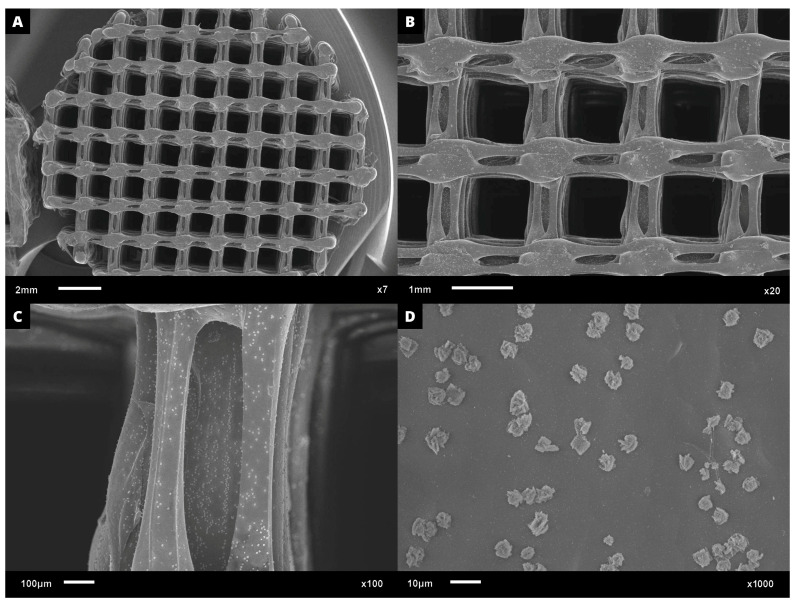
SEM microphotographs with approximation of: (**A**) 7×; (**B**) 20×, (**C**) 100×, and (**D**) 1000×, proving the L929 cell adhesion on the surface of PHB/BC 0.50% scaffolds after 3 days culture.

Regarding cell adhesion assay, the cell–scaffold interaction was examined following the cultivation of L929 cells on PHB/BC scaffolds. Following a three-day culture, SEM images were obtained. Figure 8A–D demonstrates a representative sample PHB/BC 0.50%. It is possible to observe that the blend greatly supports cell adhesion, indicating the non-cytotoxicity of the scaffolds and the provision of cell anchorage sites.

## 4. Conclusions

The PHB/BC-based scaffolds were obtained through 3D printing using filaments containing micronized BC. Among the different mechanochemical processes, BC micronized at 20 Hz showed the best properties in terms of granulometry and homogeneity for obtaining biocomposite filaments. In addition, BC micronized at 20 Hz presented a balance between the crystalline and amorphous portions verified via XRD analysis. This is an important characteristic, since BC performs the function of a reinforcing agent but can simultaneously be degraded as tissue regeneration occurs. TGA, FTIR, and SEM measurements confirmed that there is a reduction in the crystalline portion of BC, and that micronized BC at 20 Hz maintained the structure of the fibers and exhibited an insignificant decrease in thermal stability, considering the melting point of PHB for application in 3D printing. The production process of the biocomposite filaments resulted in homogeneous structures based on PHB/BC without cracks or breaks, which allows them to be used in 3D printing. The physical–chemical characterization, cytotoxicity, and cell adhesion evaluation carried out in the present study are sufficient to preliminarily validate that biocomposite filaments have the potential to be applied in tissue engineering. Obtaining these filaments also stands out, as BC industrial scraps were used. These new processes and products add value to them, as they are aligned with the concepts of circular economy, upcycling, and sustainability.

## Figures and Tables

**Table 1:** Crystallinity indices (*CI*) determined by Segal and peak deconvolution methods for bacterial cellulose (BC) scraps and BC micronized at 10, 20, and 30 Hz, respectively.

Method	Bacterial Cellulose			
	Scraps	10 Hz	20 Hz	30 Hz
Segal	0.99	0.89	0.81	0.27
Peak deconvolution	0.99	0.80	0.69	0.32

**Table 2:** Mean size and standard deviation of bacterial cellulose (BC) micronized at 10, 20, and 30 Hz.

BC Samples	Average Diameter (µm)		
	D10	D50	D90
10 Hz	33.3 ± 6.4	109.9 ± 18.6	276.1 ± 87.5
20 Hz	7.8 ± 0.7	53.6 ± 3.5	123.8 ± 12.7
30 Hz	6.4 ± 1.8	41.5 ± 12.6	74.9 ± 24.4

**Table 3:** The surface area of bacterial cellulose (BC) micronized at 10, 20, and 30 Hz.

BC Samples	Surface Area (m^2^ g^−1^)
10 Hz	1.47
20 Hz	1.59
30 Hz	1.20

**Table 4:** Degree of polymerization (DP) of bacterial cellulose (BC) scraps and their micronization at 10, 20, and 30 Hz.

Samples	Average Flow Time (s)	Degree of Polymerization (DP)
Solvent + water	31	-
Scraps	977.33	1576.28
BC 10 Hz	798.33	1387.87
BC 20 Hz	380.33	845.12
BC 30 Hz	83	207.97

## Data Availability

The data presented in this study are available on request from the corresponding authors.

## Supplementary Materials

The following supporting information can be downloaded at: https://www.mdpi.com/article/10.3390/jfb14090464/s1, Figure S1: Changes of the maximum decomposition temperature of PHB/BC related to the BC composition. Data obtained from Figure 2.
