# Supplementary material for: 3D Filaments Based on Polyhydroxy Butyrate—Micronized Bacterial Cellulose for Tissue Engineering Applications
Source: J Funct Biomater. 2023 Sep 9;14(9):464. doi: 10.3390/jfb14090464 (PMC10531805; doi:10.3390/jfb14090464)

Supplementary Material

Figure S1. Changes of the maximum decomposition temperature of PHB/BC related to the BC composition. Data obtained from Figure 2.

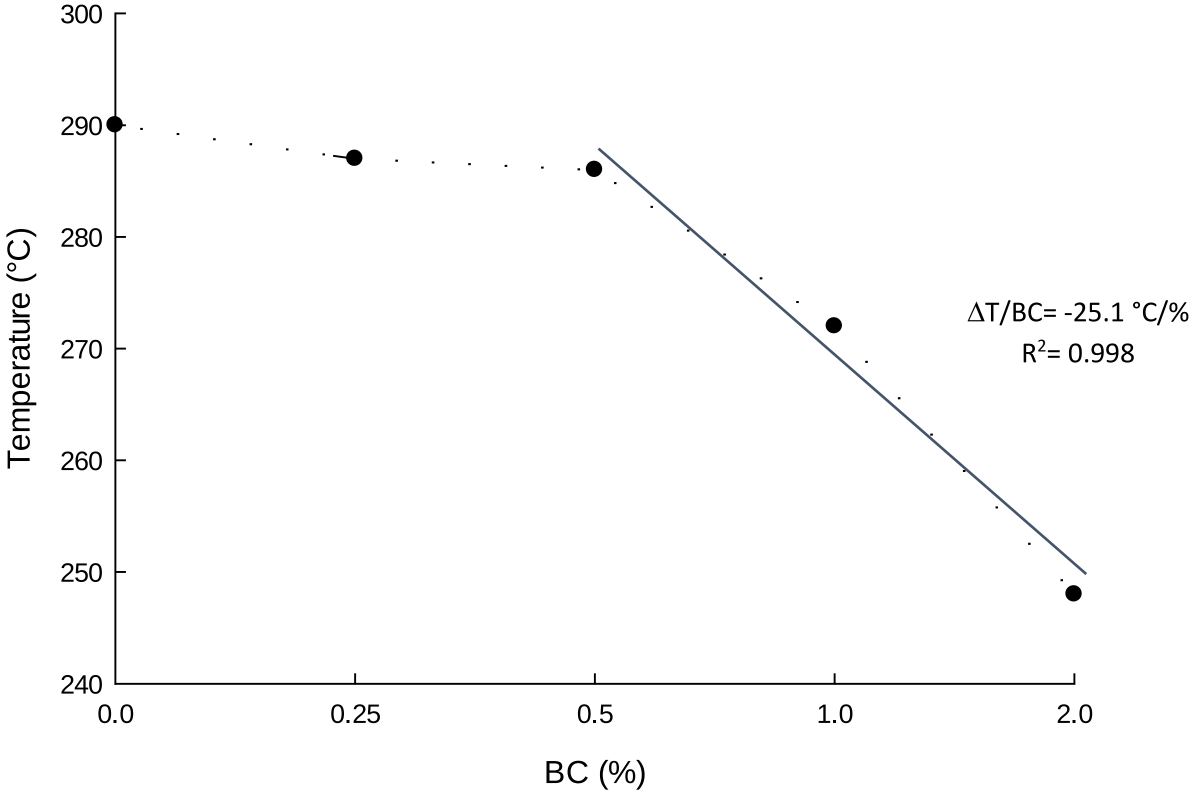

Supplement: Supplementary file 1 [file jfb-14-00464-s001.zip › jfb-2537522-supplementary.pdf]
